# Genome-Wide Association Study and Identification of Candidate Genes for Nitrogen Use Efficiency in Barley (*Hordeum vulgare* L.)

**DOI:** 10.3389/fpls.2020.571912

**Published:** 2020-09-04

**Authors:** Sakura D. Karunarathne, Yong Han, Xiao-Qi Zhang, Gaofeng Zhou, Camilla B. Hill, Kefei Chen, Tefera Angessa, Chengdao Li

**Affiliations:** ^1^Western Barley Genetics Alliance, College of Science, Health, Engineering and Education, Murdoch University, Perth, WA, Australia; ^2^Western Australian State Agricultural Biotechnology Centre, Murdoch University, Perth, WA, Australia; ^3^Department of Primary Industries and Regional Development, Government of Western Australia, Perth, WA, Australia; ^4^SAGI West, Faculty of Science and Engineering, Curtin University, Perth, WA, Australia

**Keywords:** nitrogen, barley, genome-wide association study, marker trait associations, candidate genes, yield

## Abstract

Nitrogen (N) fertilizer is largely responsible for barley grain yield potential and quality, yet excessive application leads to environmental pollution and high production costs. Therefore, efficient use of N is fundamental for sustainable agriculture. In the present study, we investigated the performance of 282 barley accessions through hydroponic screening using optimal and low NH_4_NO_3_ treatments. Low-N treatment led to an average shoot dry weight reduction of 50%, but there were significant genotypic differences among the accessions. Approximately 20% of the genotypes showed high (>75%) relative shoot dry weight under low-N treatment and were classified as low-N tolerant, whereas 20% were low-N sensitive (≤55%). Low-N tolerant accessions exhibited well-developed root systems with an average increase of 60% in relative root dry weight to facilitate more N absorption. A genome-wide association study (GWAS) identified 66 significant marker trait associations (MTAs) conferring high nitrogen use efficiency, four of which were stable across experiments. These four MTAs were located on chromosomes 1H(1), 3H(1), and 7H(2) and were associated with relative shoot length, relative shoot and root dry weight. Genes corresponding to the significant MTAs were retrieved as candidate genes, including members of the asparagine synthetase gene family, several transcription factor families, protein kinases, and nitrate transporters. Most importantly, the *high-affinity nitrate transporter 2.7* (*HvNRT2.7*) was identified as a promising candidate on 7H for root and shoot dry weight. The identified candidate genes provide new insights into our understanding of the molecular mechanisms driving nitrogen use efficiency in barley and represent potential targets for genetic improvement.

## Introduction

Nitrogen is one of the most important macro nutrients required for vegetative and reproductive development of crops, which is naturally available in soil yet applied exogenously to improve yield. However, only about 30–50% proportion of this exogenous N is absorbed by plants while the rest is leached into the environment leading to water, soil, and air pollution and is a contributor to global warming (Glass et al., 2002; Anbessa and Juskiw, 2012; Chien et al., 2016). In addition, application of exogenous N increases production costs. Improving nitrogen use efficiency (NUE) therefore requires more attention. Nitrogen use efficiency is generally defined as the grain yield per unit of N available (Moll et al., 1982). There are two major approaches to improving NUE, (1) conventional breeding techniques and (2) transgenic breeding approaches. There is strong evidence in rice, wheat, and canola to support conventional breeding methods for NUE improvement under various regimes of N availability (Kessel et al., 2012; Kowalski et al., 2016; Neeraja et al., 2019; Stahl et al., 2019). Similarly, overexpression and modification of genes through various transgenic approaches have led to NUE improvement in rice and wheat (Hu et al., 2018; Wang W. et al., 2018). Null mutations in ARE1 gene in rice improved NUE and yield under low-N (Wang Q. et al., 2018).

Barley is a major raw ingredient for the malting and brewing industries and is widely used as a livestock feed with a small yet increasing proportion entering the human consumption market. Due to its diploid nature, it is a good genetic model for other cereal crop species in the *Triticeae* tribe (Poourkheirandish and Komatsuda, 2007). It has become the subject of extensive quantitative trait loci (QTL) analysis targeting important agronomic and morphologic traits such as plant height, heading date, 1,000 grain weight, grain size and yield among others (Kindu et al., 2014; Pauli et al., 2014; Watt et al., 2019). However, only a handful of studies have detected QTL for yield of plants grown under different N treatments (Mickelson et al., 2003; Kindu et al., 2014). Recently, 15 QTLs related to NUE and its component traits such as N harvest index, N utilization of grains and aboveground biomass, and N uptake efficiency were identified in a Prisma × Apex mapping population (Kindu et al., 2014). However, most of these studies are limited by low genetic diversity, low marker density, and small population sizes.

Nitrogen use efficiency is a complex polygenic trait making its genetic dissection challenging. Genome-wide association studies (GWAS) are a common approach to dissect the genetic basis of complex traits (Risch and Merikangas, 2007) and have been used successfully to identify genomic regions contributing to numerous traits in many crop species (Wang et al., 2012; Monostori et al., 2017; Hazzouri et al., 2018). In barley, complex traits such as drought resistance, salt tolerance, and frost tolerance have been the focus of GWAS (Pasam et al., 2012; Visioni et al., 2013; Hazzouri et al., 2018; Jabbari et al., 2018; Pham et al., 2019; Mwando et al., 2020). GWAS have been conducted in wheat and rice to investigate NUE (Monostori et al., 2017; Tang et al., 2019) but not yet in barley. Hydroponic screening is an effective approach for nutrient-related studies and can accurately reflect N and P uptake efficiency traits (Liu et al., 2017).

Genes responsible for potential NUE improvement have been extensively studied in rice and wheat (He et al., 2015; Wang W. et al., 2018; Hu et al., 2019; Zhang et al., 2019). Ammonium (AMT) and nitrate (NRT1/NRT2) transporters were found to play important roles in N uptake and transport. Overexpression of *OsNRT1.1* and *OsNRT2.1* in rice increased NUE, grain yield, and plant growth (Huang et al., 2017; Wang W. et al., 2018). In addition, an amino acid biosynthesis gene *alanine aminotransferase* incorporated from barley (*HvAlaAT*) into rice increased yields considerably under low-N treatment (Shrawat et al., 2008; Selvaraj et al., 2017). Several transcription factors and protein kinases were reported to be involved in the plant N regulatory network (Yang et al., 2014; Goel et al., 2018; Hsieh et al., 2018). Many studies suggest that manipulation of genes for primary and secondary N assimilatory pathways is also an effective strategy to improve NUE (Martin et al., 2006; Pathak et al., 2008; Pathak et al., 2011).

In the present study, a hydroponics screen was carried out using a set of 282 genetically diverse barley accessions under different N concentrations to determine the genotypic performance under suboptimal N conditions. GWAS was conducted using single nucleotide polymorphism (SNP) and diversity arrays technology (DArTseq) markers to detect the significant marker trait associations with NUE related traits such as shoot length (plant height), root length, shoot dry weight, root dry weight, number of tillers, and number of leaves at seedling stage. Several candidate genes were identified that can be validated in further experiments to improve the NUE of barley.

## Materials and Methods

### Plant Materials and Experimental Design

A set of 282 barley accessions (listed in **Supplementary Table S1**) was used in the present study which represents a subset of a larger worldwide barley panel (Hill et al., 2019a). Comprising of germplasm from Africa, Asia, Australia, Europe, Middle East, North and South America including two-rowed (87%) and six-rowed (13%) head types with spring, winter, and facultative growth habits. They were grown hydroponically under two N treatments in controlled conditions at Murdoch University, Western Australia in 2018 and 2019 successive winter seasons. The experiment was arranged in a balanced incomplete block design with four biological replicates of each genotype, each grown in separate hydroponic containers.

### N Treatment and Phenotyping

Seeds were germinated on sterilized moist Whatman No.1 filter papers in Petri dishes. At the second leaf stage (7–10 days since germination), seedlings with uniform growth status were transplanted onto 27 L black containers. The plants were treated with a modified Hoagland nutrient solution (Han et al., 2018) containing 2 mmol/L NH_4_NO_3_, 0.4 mmol/L MgSO_4_, 0.3 mmol/L K_2_SO_4_, 0.2 mmol/L KH_2_PO_4_, 0.4 mmol/L CaCl_2_, 0.19 µmol/L CuSO_4_, 46.9 µmol/L H_3_BO_3_, 4.5 µmol/L MnCl_2_, 1 µmol/L Na_2_MoO_4_, 0.38 µmol/L ZnSO_4_, 19.9 µmol/L Fe (III)EDTA. The pH of the solution was adjusted to 5.8 ± 0.1 with NaOH.

The two N treatments, 0.2 mmol/L NH_4_NO_3_ (low-N) and 2 mmol/L NH_4_NO_3_ (optimal-N, as a control) were initiated after 7 days of seedling transplanting. The nutrient solution was continuously aerated with pumps and renewed twice a week. After 3 weeks of N treatments leaf yellowing (LY), number of leaves (LN), number of tillers (TN), root and shoot lengths (RL, SL) were recorded. Plants with uniform growth status were subsequently harvested as replicates and separated into roots and shoots and dried in the oven at 50°C for 3–4 days to obtain the dry weight (RDW, SDW).

### Calculation and Statistical Analysis

Relative number of tillers (Rtillers), relative number of leaves (Rleaves), relative root length (RRL), relative shoot length (RSL), relative root dry weight (RRDW), and relative shoot dry weight (RSDW) were expressed as percentages;

(x/y)×100

where *x* is the average of the phenotypic trait under low-N, and *y* is average of the phenotypic trait under optimal-N. The best linear unbiased estimates (BLUE) values for each trait, from four replicates and two independent experiments were used to identify the marker-trait associations. Analysis of BLUE was performed in R, and correlations between phenotypic traits across two years were calculated using IBM SPSS Statistics Version 24.

### Population Structure Analysis

The software STRUCTURE (version 2.3; Hubisz et al., 2009) was used to determine the underlying population structure in the diversity panel. Genotypes of the accessions were imported into STRUCTURE. Ten independent structure runs were performed with 30,543 DArTseq and SNP markers applying the admixture model. A burn-in period of 5,000 iterations followed by 5,000 Markov Chain Monte Carlo iterations was set for accurate parameter estimates. The number of populations (K) was set from 1 to 10. K was determined based on the method described by Evanno et al. (2005).

### Linkage Disequilibrium Analysis

Genome-wide LD (linkage disequilibrium) analysis was performed using the LD function in TASSEL (version 5.0) software (Bradbury et al., 2007). The analysis comprised of squared allele frequency correlation (R^2^) and normalized coefficient of linkage disequilibrium (DPrime). The locus was considered as significant LD at p < 0.05. To estimate the LD decay, R^2^ values were plotted against the physical distance between the markers, and a LOESS (locally estimated scatterplot smoothing) curve was fitted using IBM SPSS Statistics Version 24. The interception of the LOESS curve and background LD was considered as an estimate of LD decay (Monostori et al., 2017).

### Genome-Wide Association Mapping and Haplotype Analysis

The software TASSEL (version5.0) was used to conduct association mapping of low-N tolerance in barley. A set of 30,543 SNP and DArT markers was available for GWAS in the present study (Hill et al., 2019a; Hill et al., 2019b; Hill et al., 2020; Mwando et al., 2020). DArTseq genotyping by sequencing was performed using the DArTseq platform (DArT PL, Canberra, NSW, Australia). Information on genotype, population structure, and phenotypic traits (each measured and calculated) was imported to TASSEL. Association analysis was performed using Mixed Linear Model (MLM) model;

Trait=Population structure+Marker effect+Individual+Residual.

The distribution of marker p-values across barley chromosomes were visualized as Manhattan plots (R package qqman) with chromosome position as the x-axis and −log (p value) as the y-axis. False discovery rate (FDR) correction was performed using Benjamini–Hochberg method to obtain a q-value (FDR adjusted p value) (Benjamini and Hochberg, 1995). Significant marker trait associations with q < 0.05 were selected. Markers within 10 Mb were clustered into one locus based on LD decay, and the haplotypes for each locus were defined to identify beneficial haplotypes conferring high NUE. Average RSDW of the barley genotypes was used as the key parameter to represent NUE in our study (Yang et al., 2014; Xiong et al., 2019). Phenotypic effects (*a_i_*) of selected marker loci were calculated using the average phenotypic values for accessions harboring favorable and unfavorable alleles based on average values for the given traits across 2 years (Li et al., 2016).

### Identification of the Candidate Genes

By using an in-house barley blast server BarleyVar (http://146.118.64.11/BarleyVar/) with genomic variants from 20 barley accessions, the genes containing the significant low-N tolerant marker and genes between 50 Kb upstream and downstream of the MTAs were identified as potential candidates. They were blasted against the barley reference genome at IPK Barley BLAST Server (https://webblast.ipk-gatersleben.de/barley_ibsc/) to obtain gene annotations.

## Results

### Phenotypic Evaluation of Low-N Tolerance in Barley

**Figure 1:**
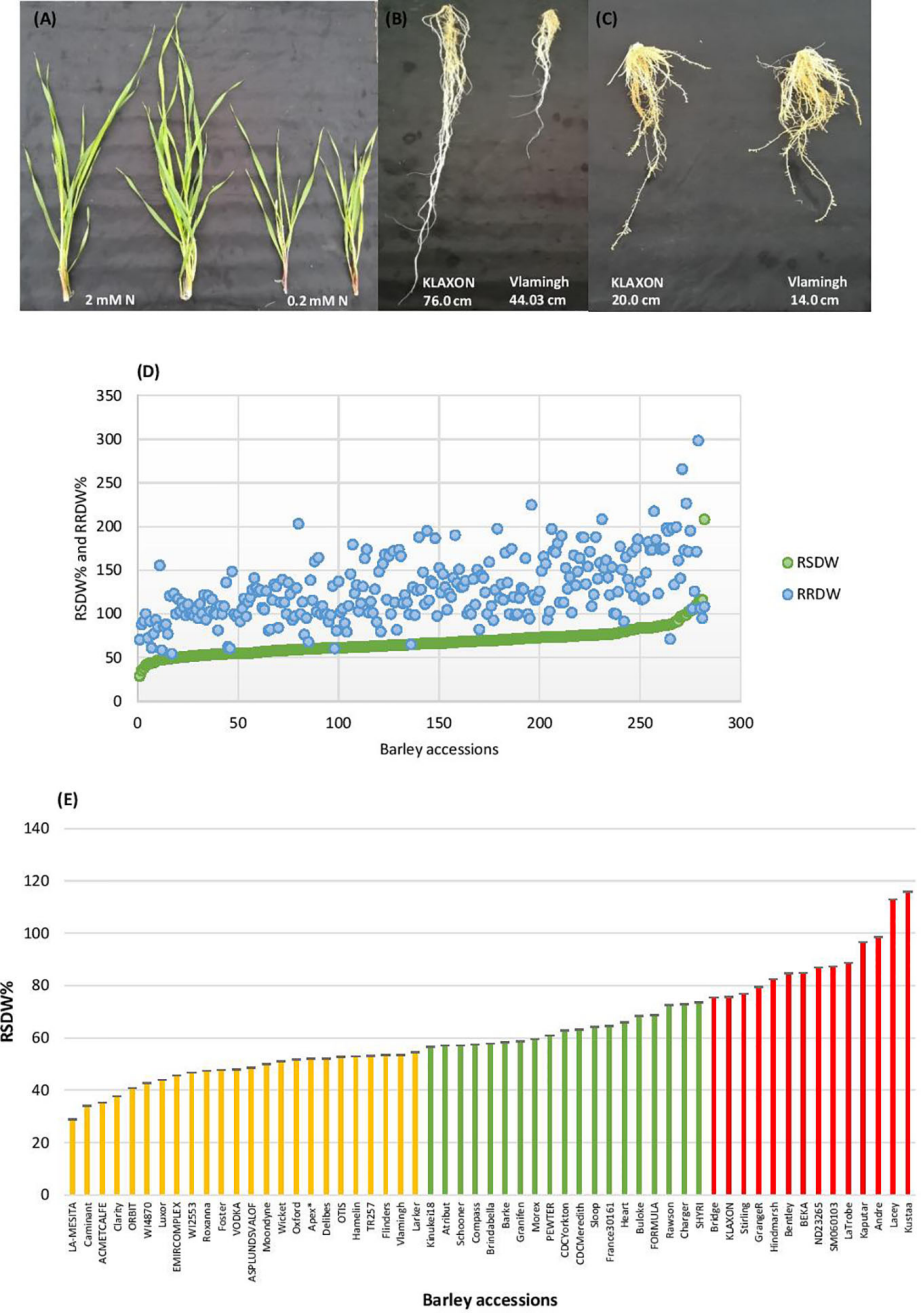
Performance of barley genotypes under low and optimal-N conditions. **(A)** Shoot growth of Vlamingh under optimal-N treatment (2 mM) and low-N treatment (0.2 mM). **(B)** Variation in root lengths of low-N tolerant KLAXON and low-N sensitive Vlamingh varieties under low-N (0.2 mM). **(C)** Root lengths of low-N tolerant KLAXON and low-N sensitive Vlamingh varieties under optimal-N (2 mM). **(D)** Distribution of relative shoot dry weight (RSDW) and relative root dry weight (RRDW) among 282 barley accessions which depicts average NUE. **(E)** Performance of barley genotypes based on RSDW under low-N. Yellow, Low-N sensitive; Green, Moderately tolerant to low-N; Red, Low-N tolerant. Apex* was used as a parental variety of a mapping population in Kindu et al. (2014).

Barley seedlings started showing clear phenotypic segregation between the two N treatments after 3 weeks. The seedlings grown under low-N were smaller with thinner stems and had pale green and narrower leaves compared to seedlings grown under optimal-N (**Figure 1A**). Measurements of root and shoot dry weights and root and shoot lengths were used to assess performance under low-N. Based on low to high relative shoot and root dry weights (RSDW and RRDW), barley accessions were categorized as low-N sensitive, moderately tolerant, and tolerant (**Table 1**). Approximately 20% of the accessions were sensitive to low-N. Low-N sensitive genotypes had an average shoot dry weight reduction of 50–70%, while the tolerant varieties had only a reduction of 25% under low-N treatment compared to optimal-N in both experiments (**Supplementary Table S2**). Relative root dry weight showed a wide range of variation among the genotypes under low-N where approximately 20% of accessions showed a 10–50% reduction and the others with an average increase of 60% compared to those under optimal-N. The population average for RRDW was around 150%. Low-N tolerant varieties had 50–100% longer roots than sensitive varieties under low-N supply (**Figure 1B**). On the other hand, all the accessions grown under optimal-N supply had relatively short root systems, mostly ranging from 25 to 50 cm (**Figure 1C**).

**Table 1:** Cut-off values for the response of barley genotypes to low-N based on relative shoot dry weight.

Category	RSDW cut-off value	Number of barley genotypes
Low-N sensitive	≤55%	58
Moderately tolerant	55–75%	167
Low-N tolerant	>75%	57

Leaf yellowing was also a prominent phenotypic trait indicating low-N sensitivity which was scored from 0 (green) to 2 (completely yellow). None of the accessions under optimal-N treatment exhibited leaf yellowing whereas more than 95% of the accessions showed leaf yellowing under low-N. In addition, the number of tillers and leaves were counted for both treatments. Tiller and leaf numbers were comparatively higher in low-N tolerant accessions than the sensitive ones under low-N. However, the number of leaves was a better indicator of low-N performance of the barley accessions only when considered together with other phenotypic traits such as dry weight.

Thus, based on the reduction of shoot dry weight under low-N, 57 varieties were identified as low-N tolerant (maintained >75% RSDW compared to the control condition (optimal-N), 58 varieties were identified as low-N sensitive (maintained ≤ 55% RSDW) (**Table 1**, **Figure 1D**). The population average for RSDW was 65%. A representative list of 57 genotypes (with the most tolerant and sensitive genotypes) which included both Australian and Canadian commercial varieties is provided in **Supplementary Table S3**; **Figure 1E**.

### Correlations Between Traits

Correlation analysis was performed for all environments with two N treatments and two experimental years for all the phenotypic traits measured (**Supplementary Table S4** and **Supplementary Figure S1**). There was a significant positive correlation between the two traits shoot dry weight (SDW) and root dry weight (RDW) (coefficient of correlation R~0.8 in both years) under low-N (**Table 2**) indicating that either of the two traits SDW or RDW can be used to score low-N tolerance at the seedling stage of barley. SDW also showed a positive correlation (R~0.65 in 2018 and R~0.7 in 2019) with leaf number.

**Table 2:** Correlation coefficients of mean values of phenotypic traits in the 2018 and 2019 seasons.

Low-N in 2018 & 2019	SL	RL	SDW	RDW	LN	TN
**SL**	–	.242^**^	.473^**^	.216^**^	.127^*^	0.058
**RL**	.246^**^	–	.388^**^	.385^**^	.323^**^	.254^**^
**SDW**	.634^**^	.394^**^	–	.775^**^	.600^**^	.476^**^
**RDW**	.351^**^	.570^**^	.802^**^	–	.578^**^	.487^**^
**LN**	.170^**^	.293^**^	.651^**^	.669^**^	–	.716^**^
**TN**	.136^*^	.230^**^	.550^**^	.604^**^	.642^**^	–

Below the diagonals are the correlation coefficients of phenotypic traits under low-N in 2018, and above the diagonals are the correlation coefficients of phenotypic traits under low-N in 2019. *Correlation is significant at the 0.05 level. **Correlation is significant at the 0.01 level. (SL, Shoot length; RL, Root length; SDW, Shoot dry weight; RDW, Root dry weight; LN, Leaf number; TN, Tiller number).

### Analysis of Population Structure and Linkage Disequilibrium Assay

**Figure 2:**
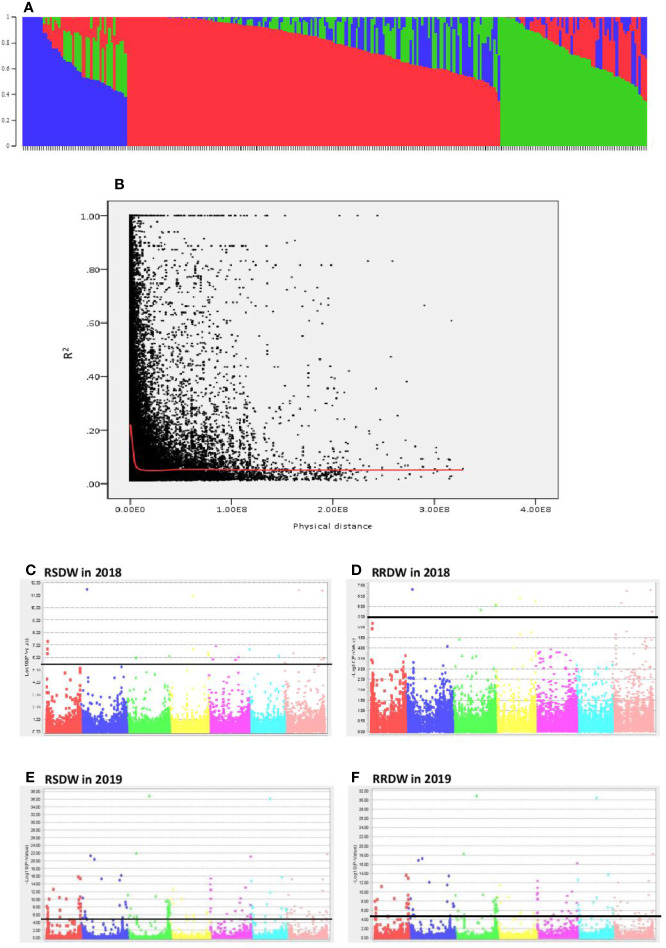
Genome wide association study of 282 barley accessions for NUE related traits under low-N. **(A)** Population structure of 282 barley accessions based on genetic diversity detected using ~30,000 markers. Three subpopulations (K = 3) are represented using different colors, with shared colors representing admixed groups. **(B)** Decay of LD of the entire barley population used. The LOESS fitting curve illustrates the LD decay (red line). **(C–F)** Manhattan plots for relative shoot dry weight (RSDW) and relative root dry weight (RRDW) in 2018 and 2019 respectively. The horizontal axis represents the seven chromosomes (1H**–**7H) of the barley genome. The vertical axis represents −log_10_(p value) of the MTAs. Horizontal black line represents the threshold value −log_10_(p) = 5.5 or −log10(p) = 4.3.

The population structure analysis assigned the 282 genotypes used in the present study to three subpopulations (K = 3) with some admixture individuals in each subpopulation. The composition of each cluster is shown in **Figure 2A** represented by three different colors. LD was calculated for the entire population by pairwise marker R^2^ for each chromosome. The mean LD decay in the 282 barley accessions was 10.6 Mb (R^2^ ~ 0.2). The LD decay of the population estimated as the intercept of the LOESS curve is given in **Figure 2B**.

### Marker-Trait Associations and the Beneficial Haplotypes for NUE

Two independent GWAS were performed using data collected under low-N and optimal-N for seven phenotypic traits and their calculated relative values in both years. The significance level of the threshold for the traits with FDR correction were *P* = 3.19 × 10^-6^ and *P* = 4 × 10^-5^ with −log10(p) values as 5.5 and 4.32, respectively in 2018 and 2019.

A total of 299 markers associated with different nitrogen use efficiency (NUE) related traits: RL, SL, RDW, SDW, Rtillers, Rleaves, RRL, RSL, RRDW, RSDW were identified from 2018 and 2019 GWAS based on FDR with q < 0.05. Only the significant associations were selected from the above marker set if the markers associated with at least two phenotypic traits under low-N. Accordingly, the list was narrowed down to 136 MTAs, and it was obvious that the majority of these markers were associated with RSDW and RRDW as two key traits of NUE (**Figures 2C–F**).

**Figure 3:**
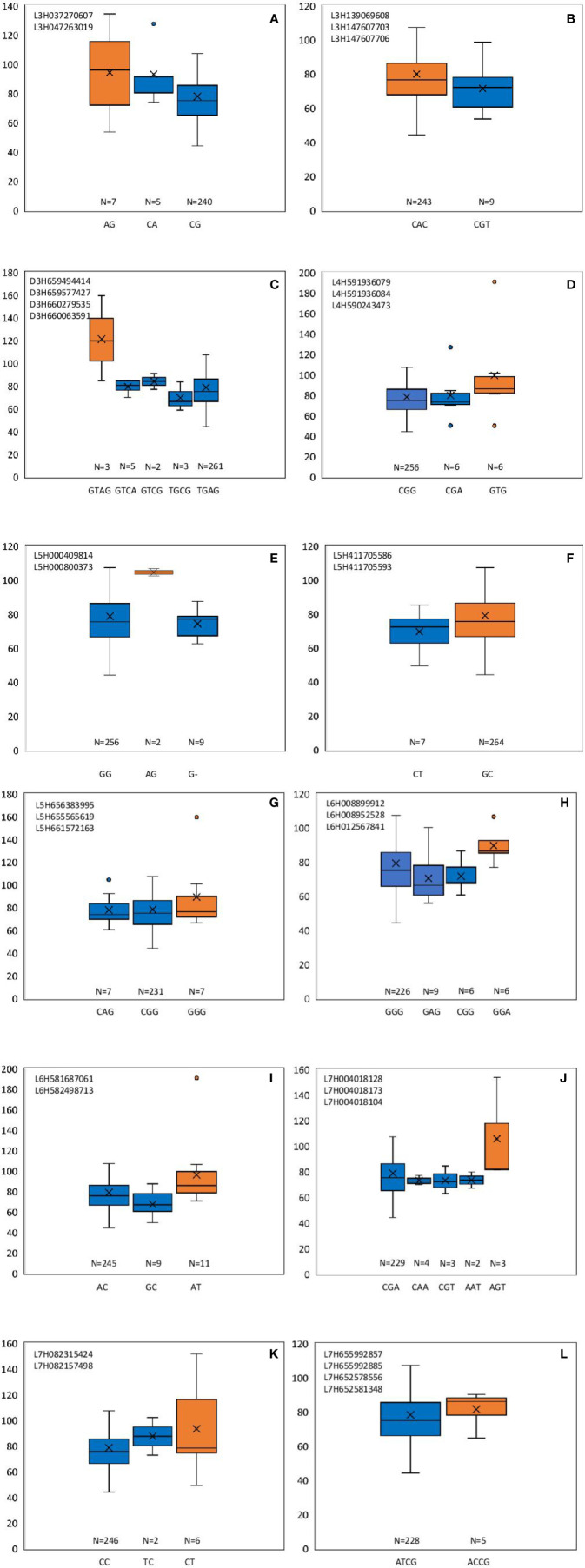
Haplotypes defined for the most significant MTAs identified in 2018 and 2019. **(A–L)** Most beneficial haplotype conferring high NUE per locus is highlighted in orange. The y-axis indicates average NUE, and the x-axis indicates the haplotype. N is the number of barley accessions represented by each haplotype.

From these significant MTAs, associated markers flanking a region of 10 Mb, based on LD decay were clustered as one MTA region which reduced the number of MTA regions to 66 (15 MTAs in 2018 and 51 MTAs in 2019) (**Supplementary Tables S5** and **S6**). The 15 MTAs identified in 2018 were on chromosomes 1H (1), 2H, (1), 3H (2), 4H (2), 5H (1), 6H (2), and 7H (6). Similarly, there were eight MTAs on chromosomes 1H, nine on 2H, 12 on 3H, three on 4H, six each on 5H and 6H and seven on 7H identified in 2019. Haplotypes were defined for each identified locus in both experiments and the NUE was averaged for each haplotype (**Supplementary Table S5**). Haplotype conferring high NUE was considered as the beneficial haplotype and subsequently as the most significant locus. For instance, MTA 44 flanked by markers L6H581687061, L6H582498713 had three main haplotypes as “AC, GC, AT” with 79, 67, and 96% average NUE respectively (**Figure 3I**) where haplotype “AT” was clearly the most beneficial haplotype at this locus. The 12 most significant loci based on the haplotype analysis are presented in **Figure 3**. Among them, there were four stable MTAs (**Supplementary Figure S2**) repeatedly identified in both years for one or more traits: RSDW, RRDW, and RSL on chromosomes 1H (1), 3H (1) and 7H (2). For instance, markers L3H147608174 and L3H147608182, L3H147607703, and L3H147607706 on chromosome 3H were identified for RSDW and RRDW in both years (**Supplementary Figure S2**). For further analysis, these stable MTAs repeated in both years were considered.

Phenotypic effect (*a_i_*) was calculated for the marker loci contributing to the above four stable MTAs, and alleles for trait improvement were identified (**Table 3**). Alleles with positive phenotypic effects that led to the increase of RSDW, RRDW, RSL were identified as favorable alleles whereas marker alleles with negative effects were identified as unfavorable. Marker L7H004016220 (tightly linked with markers L7H004018128 and L7H004018104) had positive effects on RSDW and RRDW. Taken together with other phenotypic data obtained throughout the study, NUE of the genotypes with favorable alleles for RSDW and RRDW was moderate to high, and those with unfavorable alleles were low. For instance, genotypes Hindmarsh, KLAXON, and Lacey discovered as high NUE from the present study had favorable alleles for the markers L3H147608174, L3H147608182, and L7H004018104 (**Table 3** and **Supplementary Table S3**). Also, high nitrogen use efficient cultivars Lacey, Hindmarsh, and Bentley had favorable alleles for L1H017313985.

**Table 3:** Favorable alleles, phenotypic effects (a_i_*) and the number of accessions.

Marker	p value	−log10(p) value	Trait	Chr	Ref allele	Alt allele	Fav allele	a*_i_* * value	No of accessions with fav allele
L1H017313420	5.12E-07	6.29	RSDW	1	–	T	–	1.33	202
L1H017313985	2.09E-07	6.68	RSDW	1	T	C	T	0.29	213
L3H147608174	1.04E-06	5.98	RSDW	3	A	G	A	0.74	240
L3H147608182	1.05E-06	5.98	RSDW	3	G	A	G	0.73	240
L7H004016220	2.86E-06	5.54	RSDW	7	T	C	T	0.21	244
L7H004016220	6.81E-04	3.17	RRDW	7	T	C	T	0.56	244
L7H004018104	4.31E-06	5.37	RSDW	7	A	T	A	0.27	238
L7H004018128	6.54E-07	6.18	RRDW	7	C	A	C	0.52	243
L7H640049188	8. 41E-09	8.08	RSL	7	T	G	T	0.12	263

Ref allele is the reference allele, Alt allele is the alternate allele and Fav allele is the favorable allele, a_i_* is the phenotypic effect. RSDW, relative shoot dry weight; RRDW, relative root dry weight; RSL, relative shoot length.

### Candidate Genes Associated With Tolerance to Low-N

A total of 140 candidate genes (**Supplementary Table S5**) were identified by aligning the significant marker positions from the 66 MTAs in the recently annotated barley reference genome (http://146.118.64.11/BarleyVar/& https://webblast.ipk-gatersleben.de/barley_ibsc/). There were 25 genes on chromosome 1H, 11 on 2H, 39 on 3H, 7 on 4H, 16 each on 5H and 6H, and 26 on 7H. Out of the 140 candidate genes located on seven chromosomes, 93 could be excluded due to the weaker associations with the QTL region or with annotated function less related to NUE. The majority of the rest 47 genes located very close to the significant markers, whereas a few of them contained the marker itself providing strong evidence for candidate gene identification (**Table 4**). They belonged to several functional categories, namely, protein kinases, transcription factors or other stress-related candidates while some of them had unknown functions. Most importantly, one promising candidate gene encoding high-affinity nitrate transporter 2.7 was identified on chromosomes 7H with close proximity to the marker C7H600147760, through BLAST-based annotation. In addition, some other strong candidate genes such as leucine-rich repeat receptor-like protein kinase family protein gene (7H) and an F-box domain-containing protein gene (7H) aligned strongly with two of the most significant markers L7H004018173 and L7H640049188, respectively (**Table 4**). Genes encoding asparagine synthetase 2 (D1H549784675) and sodium coupled neutral amino acid transporter (L6H502984487) might also be useful in low-N tolerance. Protein kinase superfamily protein and serine/threonine-protein kinase were repeatedly identified at different chromosomes exhibiting their involvement in low-N tolerance.

**Table 4:** List of candidate genes for potential NUE improvement in barley.

Trait	Gene	Chr	Marker	Start–End	Annotation
Rleaves, RRL, Rtillers, RSDW	HORVU1Hr1G033980	1	L1H217665224, L1H217665227, L1H217665220	218377991–218379496	Transcription factor bHLH140
Rleaves, RSL, RRDW, RSDW	HORVU1Hr1G024420	1	L1H112101141	111584928–111590266	Asparagine–tRNA ligase
Rleaves, Rtillers, RRDW, RSDW	HORVU1Hr1G092130, HORVU1Hr1G092110	1	D1H549784675, D1H549810050	549783754–549787246, 549769608–549775894	WRKY DNA-binding protein 23, Asparagine synthetase [glutamine-hydrolyzing] 2
RSDW, RRDW	HORVU1Hr1G007930	1	L1H017313985, L1H017313420, L1H017313264	17203146–17207877	Receptor kinase 1
Rtillers, RRDW, RSDW	HORVU1Hr1G094990		L1H557218707	556905146–556910542	Protein kinase superfamily protein
Rleaves, RRL, Rtillers, RRDW, RSDW, RSL	HORVU2Hr1G036250	2	L2H160145958	159918998–159919803	Zinc finger A20 and AN1 domain-containing stress-associated protein 9
					
RRDW, RSDW	HORVU2Hr1G023180	2	C2H68835493, C2H68835922, C2H68836383	68833703–68836778	Protein FLOWERING LOCUS T
RSDW, RRDW, Rleaves, Rtillers	HORVU3Hr1G116150	3	L3H695384685, L3H695382868	695340362–695354154	BnaA01g30480D protein
Rleaves, Rtillers, RRDW, RSDW, Rleaves, Rtillers	HORVU3Hr1G000420	3	L3H001082150	1113533v1160560	Serine/threonine-protein kinase ATM
RRDW, RSDW	HORVU3Hr1G098810, HORVU3Hr1G098820	3	D3H660063591	660060877–660062773, 660072924–660073906	FAR1 family, putative, Glutathione S-transferase family protein
RRDW, RSDW	HORVU3Hr1G095880	3	D3H650981422	651020620–651022321	NAC domain protein
RRDW, RSDW	HORVU3Hr1G096720	3	D3H654302848	654302473–654309516	Unknown function
RRDW, RSDW	HORVU3Hr1G095090	3	D3H649054975	649010625–649043473	MADS-box transcription factor family protein
RRDW, RSDW	HORVU3Hr1G098610	3	D3H659494414	659501460–659502707	Leucine-rich repeat receptor-like protein kinase family protein
RRDW, RSDW	HORVU3Hr1G098920	3	D3H660279535	660282090–660285460	Succinate dehydrogenase subunit 4
RRDW, RSDW	HORVU3Hr1G030580	3	L3H147607703, L3H147607706, L3H147608174, L3H147608182	147599908–147613052	Replication protein A 32 kDa subunit B
Rleaves, Rtillers, RSDW	HORVU3Hr1G015740	3	L3H037270607	37279795–37282055	Transcription factor GTE9
Rtillers, RRDW, RSDW	HORVU4Hr1G073520	4	L4H590243473	590208225–590208651	Auxin-induced protein 5NG4
RRDW, RSDW	HORVU4Hr1G012820	4	L4H043542131	43540550–43542049	Disease resistance protein RPM1
RRDW, RSDW	HORVU4Hr1G012940	4	L4H043655413, L4H043655390	43679183–43679901	Unknown function
Rtillers, Rleaves, RSDW	HORVU4Hr1G035220	4	L4H258403614	257868981–257870162	Unknown function
RSDW, RRDW	HORVU4Hr1G064820	4	D4H542586605	542585648–542588347	Protein NRT1/PTR FAMILY 8.3
Rleaves, Rtillers, RRDW, RSDW, RSL	HORVU5Hr1G000120	5	L5H000409814	441661–441901	Sucrose transporter 4
Rleaves, Rtillers, RRDW, RSDW	HORVU5Hr1G005910	5	L5H009590416, L5H009590448, L5H009590411, L5H009590386, L5H009591047, L5H009590434	9599498–9600567	Late embryogenesis abundant protein D-34
RRDW, RSDW	HORVU5Hr1G052600	5	L5H411705586, L5H411705593	411705218–411709576	Undescribed protein
RRDW, RSDW	HORVU5Hr1G052590	5	L5H411705586, L5H411705593	411703643–411704235	B protein
Rleaves, Rtillers, RRDW, RSDW	HORVU5Hr1G087040	5	L5H577696873	577658366–577662325	Serine/threonine-protein kinase
RSDW, RRDW	HORVU5Hr1G119650	5	L5H656383995	656387855–656393736	Ethylene receptor
RSDW	HORVU6Hr1G001200	6	L6H004021832, L6H004021834	3952786–3976837	Receptor-like protein kinase 1
RSDW	HORVU6Hr1G069690	6	D6H484044374	484042599–484043907	Basic helix-loop-helix (bHLH) DNA-binding superfamily protein
Rleaves, RRL, Rtillers, RRDW, RSDW, RSL	HORVU6Hr1G051370	6	L6H312413768	312426934–312427189	Unknown function
Rleaves, Rtillers, RRDW, RSDW	HORVU6Hr1G051300	6	L6H312323946	312052625–312054058	Glutathione reductase
Rleaves, Rtillers, RRDW, RSDW	HORVU6Hr1G072350	6	L6H502984487	502959206–502968928	Sodium-coupled neutral amino acid transporter 1
RRDW, RSDW	HORVU6Hr1G094720	6	L6H581687061	581629386–581632269	Unknown function
RRDW, RSDW	HORVU6Hr1G094650	6	L6H581687061	581552566–581556341	Zinc finger CCCH domain-containing protein 19
RRDW, RSDW	HORVU6Hr1G003990	6	L6H008899912	8892068–8893512	Undescribed protein
RRDW, RSDW, Rtillers	HORVU7Hr1G088790	7	L7H539446645	539205606–539206966	Ethylene-responsive transcription factor 11
RRDW, RSDW	HORVU7Hr1G105780	7	L7H617670049, L7H617670050	617669550–617670231	Undescribed protein
RSDW	HORVU7Hr1G098550	7	C7H600147760	598424105–598497081	High-affinity nitrate transporter 2.7
Rleaves, Rtillers, RRDW, RSDW	HORVU7Hr1G095980	7	L7H585644789, L7H585644784	585668738–585670356	UDP-Glycosyltransferase superfamily protein
Rleaves, Rtillers, RRDW, RSDW	HORVU7Hr1G036070	7	L7H082315424	82159514–82315426	12-oxophytodienoate reductase 2
Rleaves, Rtillers, RRDW, RSDW	HORVU7Hr1G122350	7	L7H655992857	655997675–655999540	2-oxoglutarate (2OG) and Fe (II)-dependent oxygenase superfamily protein
RSDW, RRDW	HORVU7Hr1G122800	7	L7H655992885	657007676–657010806	Thionin-like peptide
Rtillers, RSL	HORVU7Hr1G120820	7	L7H652578556, L7H652581348		Unknown protein
RSDW, RRDW, Rleaves	HORVU7Hr1G002010	7	L7H004018128, L7H004018173, L7H004018104, L7H004016220	4019218–4024122	Leucine-rich repeat receptor-like protein kinase family protein
RRDW, RSDW, RSL	HORVU7Hr1G114730	7	L7H640047456, L7H640047457, L7H640047480, L7H640049188	640048567–640051420	F-box domain containing protein

Genes containing the significant markers or in close proximity to the markers are identified and listed as candidate genes. RSDW, relative shoot dry weight; RRDW, relative root dry weight; RSL, relative shoot length; RRL, Relative root length; Rtillers, Relative number of tillers; Rleaves, Relative number of leaves.

## Discussion

Nitrogen use efficiency is tightly related with morphological and agronomic traits such as tiller number, shoot length (plant height), root length, shoot and root dry weights, and most importantly yield (Beatty et al., 2010; Safina, 2010; Gao et al., 2017; Ghoneim et al., 2018). As yield components such as grain size and grain number per spike are difficult to determine in a larger population, our work focused mainly on the shoot and root dry weights which were promising indicators for NUE in recently conducted research (Yang et al., 2014; Xiong et al., 2019). Positive correlation between shoot and root dry weights (R~0.8 in both years) in our study confirmed that either of the two traits can be used to score low-N tolerance. We conducted a hydroponic screen of 282 barley accessions to observe phenotypic changes under low-N treatment. Several studies have been carried out so far in wheat, maize, and barley using hydroponics as a screening method (Sun et al., 2013; Yang et al., 2014; Liu et al., 2017; Ranjitha et al., 2017; Xiong et al., 2019) because of the ability to supply precise amounts of nutrients and easy observation of root characteristics. Furthermore, it has been reported that the root system architecture-related traits studied in hydroponic experiments have a positive correlation with nutrient (mainly N and P) uptake efficiency traits (Liu et al., 2017). The six quantitative traits SDW, RDW, RL, SL, TN, LN and the qualitative trait LY scored in this study exhibited distinct changes between different genotypes under low-N. However, dry weight should be the most important parameter related to growth at the seedling stage (Yang et al., 2014). Hence, we used the relative dry weights (ratios of low-N to optimal-N) of shoots and roots to investigate barley’s tolerance to low-N.

Root architecture parameters, such as root length and lateral root number were also prominent characteristics in all genotypes under low-N than that of optimal-N which implies their response to low-N by increasing their root surface area. The results reported by Tian et al. (2014) reinforce our study, finding an increase in lateral root elongation in Arabidopsis under low $NO_{3^{}}^{-}$. Similarly, exogenous $NO_{3^{}}^{-}$ stimulated lateral root elongation in maize (Postma et al., 2014; Zhan and Lynch, 2015). Assessing maize recombinant inbred lines (RILs), Li et al. (2015) reported that 70% of the QTLs for NUE, NUpE, and NUtE overlapped with QTLs controlling seedling root traits, suggesting a large contribution of morphological root traits to NUE. When available N is limited, the ability to absorb N is more important which is more related to root morphology, whereas N utilization becomes important than N absorption when available N is not limiting (Monostori et al., 2017), suggesting the well-developed root systems of low-N tolerant varieties facilitate more N absorption and improve plant growth under N limiting conditions. Specifically, the low-N tolerant varieties Granger, KLAXON, and Bridge (**Supplementary Table S3**) developed very large and long root systems with ~70–80% increase in RRDW compared to low-N sensitive varieties such as Foster, Vodka, and Wicket. Besides, there were a very few high responsive genotypes (RSDW ≥ 200% and RRDW ≥ 250%) suggesting a significantly high uptake of N by these plants even under low-N supply (He et al., 2017; Sigua et al., 2018). These genotypes performed well in conditions of both low and optimal-N and might be exploitable for breeding. The varieties with intermediate tolerance to low-N have a strong potential for further NUE improvement and thus require further attention in future studies.

We observed a considerable number of MTAs of which the majority related to RSDW, RRDW, Rtillers, and Rleaves. Very few of these corresponded to QTLs reported in previous studies (Kindu et al., 2014). Marker L5H411705586 associated with RSDW and RRDW was located on chromosome 5H in this study, in close proximity to the QTL for leaf weight (Lw) described by Kindu et al. (2014) both under no-N and low-N using 94 RILs of a Prisma × Apex mapping population. Kindu et al. (2014) have also reported QTLs for plant height (Ph) on chromosomes 1H and 3H under low-N. In accordance with these results, we observed L3H350883753 on chromosome 3H for RSL (similar to Ph) under low-N in our experiment. It is difficult to compare these results as the previous study has used a barley mapping population whereas we have used a natural population. Abdel-Ghani et al. (2019) reported QTLs for shoot biomass in a diverse spring barley panel on chromosomes 3H, 4H, and 7H which are in concordance with markers L3H350883746, L4H591936079, L7H082315424 identified in our study. Two other QTLs for root dry weight on chromosomes 1H and 2H and one QTL for tiller number on 7H under different-N levels overlapped with MTAs from the present study (Hoffmann et al., 2012). However, the majority of the MTAs reported in the present study have not been discovered previously to the best of our knowledge. These significant MTAs associated with NUE related traits are useful for marker-assisted selection in barley breeding programs.

Candidate genes retrieved from the significant MTAs belong to key gene families such as the asparagine synthetase (ASN) gene family, bHLH, WRKY, MADS and NAC transcription factors (TFs), protein kinases, and nitrate transporters. They have been reported as genes associated with N metabolism in maize, soybean, wheat, and Arabidopsis (Law et al., 2003; Hao et al., 2011; Curtis et al., 2018; Jiang et al., 2018). In our study, a gene encoding asparagine synthetase 2 (*HvASN2*) was identified on chromosome 1H. A functional homolog of it was reported in maize and was shown to be downregulated under low-N (Jiang et al., 2018). Asparagine is a major N storage and transport compound, and asparagine synthetase acts as an important switching enzyme in N metabolism (Canas et al., 2010). Overexpression of *ASN1* in *Arabidopsis* was shown to increase the N status of the plant and seed protein content (Law et al., 2003). *TaASN1* in wheat is the most responsive gene to N availability compared to the alternative ASN genes; *TaASN2*, *TaASN3*, *TaASN4* (Curtis et al., 2018). A marker that overlapped with HORVU1Hr1G092130, encoding a WRKY transcription factor, was identified from the present study. Similarly, *ZmWRKY36* genes in maize with similar annotations to the barley WRKY gene have been reported as signaling genes which respond to abiotic and biotic stresses including low-N (Jiang et al., 2018). WRKY TFs were highly induced under low KNO_3_ in *Brassica juncea* (Goel et al., 2018).

Several other TFs; NAC-domain protein (3H), MADS-box TF (3H), bHLH (1H and 6H) were identified in barley under low-N. *PvNAC1* and *PvNAC2* in switchgrass were identified as positive regulators of leaf senescence and NUE (Yang et al., 2015). Overexpression of *TaNAC2-5A* in wheat increased the tiller and spike number, shoot and root dry weight, grain N accumulation, and thousand-grain weight under low-N compared to high-N with ~10% yield improvement than the wild type. *TaNAC2-5A* also upregulated the expression of nitrate transporters (*TaNRT2.1, TaNPF7.1*) and assimilation genes (*TaGS2*) (He et al., 2015). Thus, this represents a good candidate gene for NUE improvement in wheat. Furthermore, rice overexpressing *OsMADS25* promoted shoot length, lateral and primary root growth under N free conditions (Zhang et al., 2018). It could also promote the accumulation of nitrate and expression of nitrate transporters under high-N (Yu et al., 2015). The expression of basic helix loop helix TF *bHLH120* in rice was also strongly induced by N deficiency (Hsieh et al., 2018).

In addition, two other candidate genes encoding leucine-rich repeat receptor-like kinases (LRR-RLKs) and one gene encoding receptor-like protein kinase 1 (RPK1) were located on barley chromosomes 3H, 7H, and 6H respectively. In *Arabidopsis*, *RPK1* was upregulated under stressful conditions; dehydration, low temperature, and high salt concentrations (Osakabe et al., 2005). This suggests that *RPK1* in barley might also respond in a similar fashion. Most of the functions of the LRR-RLKs are unknown, whereas well-characterized LRR-RLKs help in signal perception and plant growth (Osakabe et al., 2005). Expression of LRR-RLKs in response to low-N in crops has not been clearly reported.

Stress associated proteins such as A20/AN1 zinc-finger proteins regulate stress signaling in plants (Kothari et al., 2016). Dansana et al. (2014) reported improved water-deficit stress tolerance by overexpression of *OsiSAP1*, an A20/AN1 zinc-finger protein in rice. Similarly, zinc finger CCCH family genes exhibited stress-responsive expression in *Brassica rapa* under salt and drought stress (Pi et al., 2018). This allows us to speculate that gene zinc finger A20/AN1 on 2H and the gene on 6H which is zinc finger CCCH domain-containing protein may play a potential role in low-N tolerance in barley.

High-affinity nitrate transporter 2.7 (*HvNRT2.7*) was identified on chromosome 7H which is the functional homolog of *AtNRT2.7* in *Arabidopsis* (69% sequence identity). *AtNRT2.7* showed only slight variation in its expression in root under N limiting conditions yet had a leaf specific expression. It could be involved in nitrate influx to keep the balance in leaves between the amount of nitrate used for assimilation and re-absorbed for further transport (Orsel et al., 2002). Results from Chopin et al. (2007) also demonstrate that *AtNRT2.7* is expressed in leaves and necessary for seed nitrate accumulation. *Atnrt2.7* mutants had an average of 50% nitrate reduction in seeds compared to the wild type. High affinity nitrate transporter *OsNRT2.3b* has been successfully overexpressed in barley transgenic lines to improve yield and nutrient uptake balance by Luo et al. (2020) using hydroponics with a nearly similar concentration of N to our study. Another NRT2 gene *HvNRT2.1* was found to improve yield in *Arabidopsis* under low-N (Guo et al., 2020). Additionally, protein NRT1/PTR FAMILY 8.3 (*HvNRT1*) gene HORVU4Hr1G064820 was identified on chromosome 4H under low-N and optimal-N supply but with a low threshold of p < 0.0001. It may be due to the limited number of traits scored for phenotyping. However, there are several previous findings in agreement with the results revealed from our study on NRT1 as a candidate (Huang et al., 2017; Wang W. et al., 2018; Hu et al., 2019; Zhang et al., 2019). Loss of function of *OsNRT1.1A* in rice exhibited a significant decrease in plant height, panicle size, seed setting rate, grain yield (by ~80% than the wild type), whereas its overexpression led to increasing plant height, panicle size, seed number per panicle, biomass, chlorophyll content, yield (by ~60% than the wild type) under low-N (Wang W. et al., 2018). *OsNRT1.1A* was therefore identified as a gene which can improve NUE and grain yield simultaneously in rice. (Protein sequence identity between *OsNRT1.1A* and *HvNRT1* was 42%). This allows us to hypothesize these two nitrate transporters identified in barley are promising loci for NUE improvement. The other genes included in **Table 4** have very less or no information reported related to NUE or low-N stress to the best of our knowledge and provides a strong background for further functional characterization and validation.

## Conclusion

To improve nitrogen use efficiency in barley, unique genes and markers linked to low-N tolerance genes must be identified. Therefore, in the present study, we performed GWAS mapping within a set of genetically diverse barley accessions and identified significant MTAs for NUE. MTAs for RSDW, RRDW, RSL which expressed in both 2018 and 2019 were identified as stable MTAs that should be further explored for use in marker-assisted selection programs. Candidate gene pool provides a strong background for potential genetic manipulation in NUE improvement and open up avenues for further functional characterization. A high-affinity nitrate transporter 2.7 (*HvNRT2.7*) was discovered on chromosome 7H and found to associated with shoot and root dry weight. The MTAs and associated regions identified in this study are promising resources to guide further research aimed at improving and understanding NUE in barley.

## Data Availability Statement

The datasets analyzed and generated for this study are included in the article/**Supplementary Material**.

## Author Contributions

SK conducted the phenotyping experiments. SK and KC designed the hydroponics trial and performed statistical analysis. SK and GZ conducted data analysis and interpretation. CH and X-QZ generated the genotypic data. TA provided the seeds from his field experiments. CL, YH, and X-QZ conceived the project. SK drafted the manuscript with inputs from all authors.

## Funding

This project was made possible by the generous support of Murdoch University and Department of Primary Industry and Regional Development Western Australia.

## Conflict of Interest

The authors declare that the research was conducted in the absence of any commercial or financial relationships that could be construed as a potential conflict of interest.
