# Supplementary figures and images for: Genome-Wide Association Study and Identification of Candidate Genes for Nitrogen Use Efficiency in Barley (Hordeum vulgare L.)
Source: Front Plant Sci. 2020 Sep 4;11:571912. doi: 10.3389/fpls.2020.571912 (PMC7500209; doi:10.3389/fpls.2020.571912)

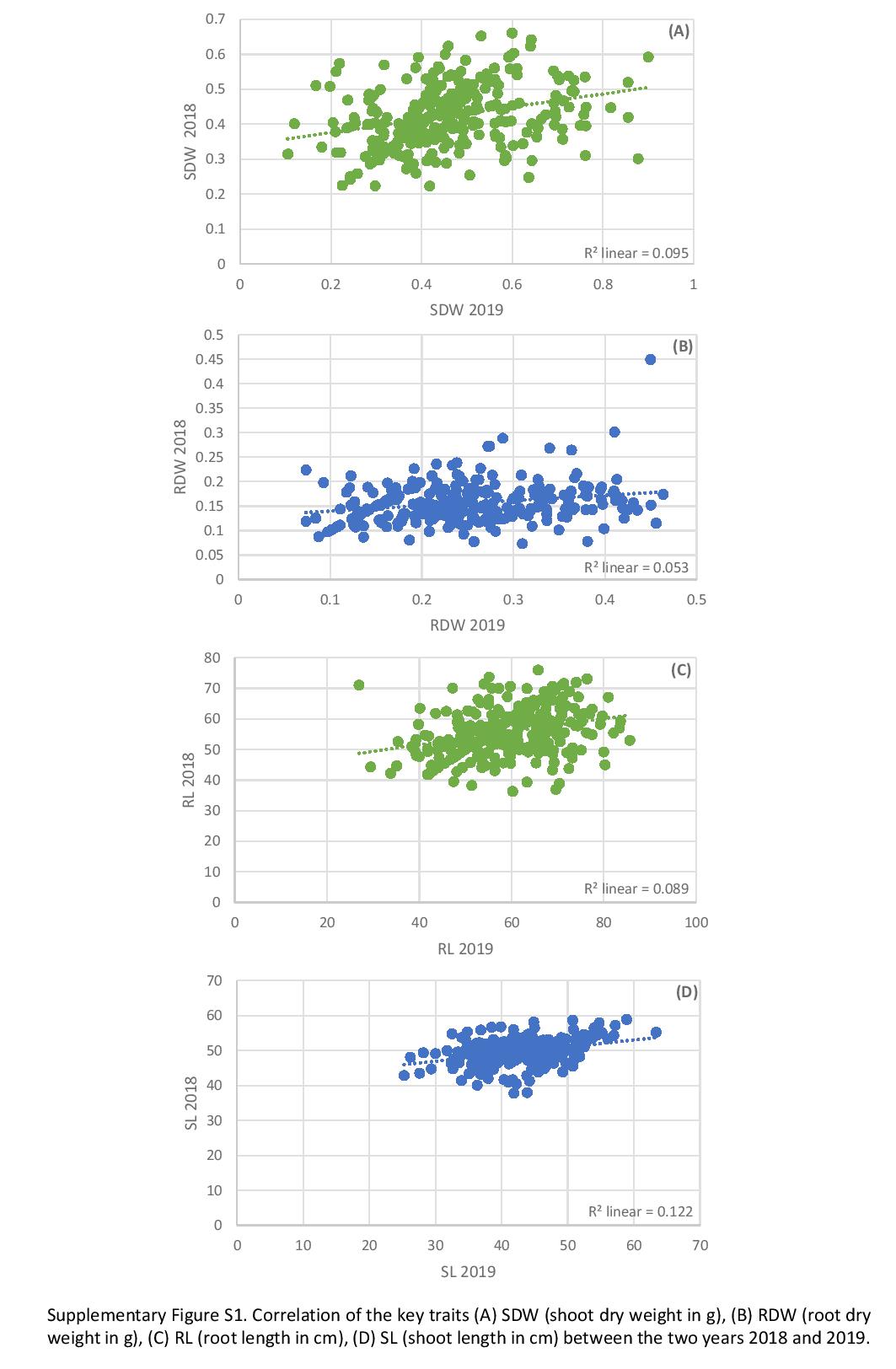

Supplement: Supplementary file 1 [file Image_1.jpeg]

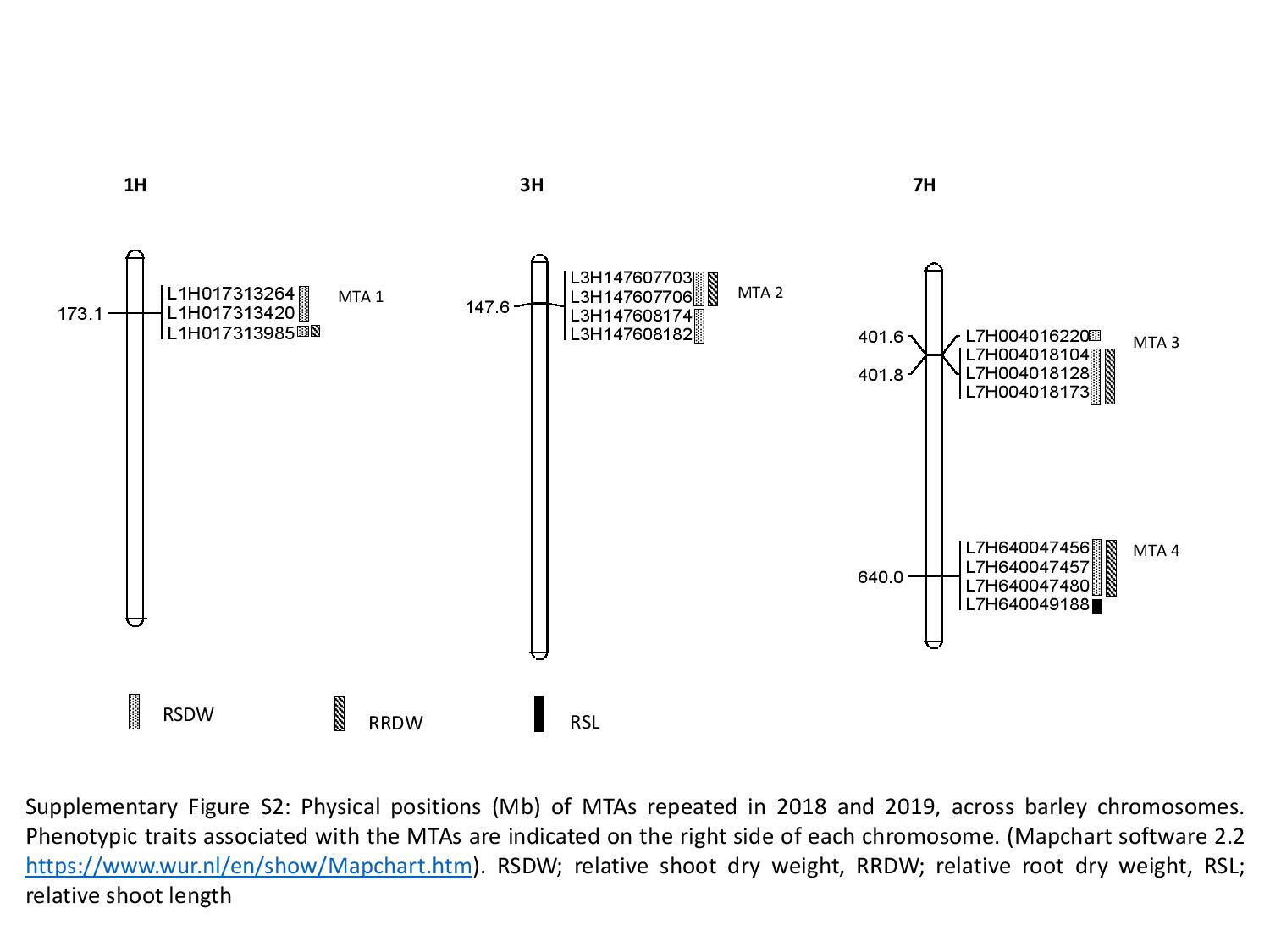

Supplement: Supplementary file 2 [file Image_2.jpeg]
